# A new method to investigate the catalytic mechanism of YhdE pyrophosphatase by using a pyrophosphate fluorescence probe

**DOI:** 10.1038/s41598-017-08368-1

**Published:** 2017-08-15

**Authors:** Qingya Shen, Hongwei Tan, Guo-wen Xing, Jimin Zheng, Zongchao Jia

**Affiliations:** 1 College of Chemistry, Beijing Normal University, Beijing, 100875 China; 2 Department of Biochemical and Molecular Science, Queen’s University, Kingston, Ontario, K7L 3N6 Canada

## Abstract

YhdE is a Maf (multicopy associated filamentation) proteins from *Escherichia coli* which exhibits pyrophosphatase activity towards selected nucleotides, although its catalytic mechanism remains unclear. Herein we used a novel fluorescence probe (4-isoACBA–Zn(II) complex) to characterize the enzymatic properties of YhdE and its mutant, establishing a new method for assaying pyrophosphatase catalytic function. Our results reveal for the first time that the new fluorescence sensor confers high sensitivity and specificity and pyrophosphate (PPi) is the direct catalytic product of YhdE. Crystal structures of a mutant in the active-site loop (YhdE_E33A) show conformational flexibility implicated in the catalytic mechanism of YhdE. ITC experiments and computational docking further reveal that Asp70 and substrate dTTP coordinate Mn^2+^. Quantum mechanics calculations indicate that YhdE hydrolysis appears to follow a stepwise pathway in which a water molecule first attacks the α-phosphorus atom in the substrate, followed by the release of PPi from the pentavalent intermediate.

## Introduction

YhdE was first identified by genome sequencing, which is located in the *mre* operon^1^ containing five genes (*mreB*, *mreC*, *mreD*, *orfE* and *orfF*). The function of YhdE (earlier known as *orfE*) was completely unknown until a homolog was found in *Bacillus subtilis*. Sharing 46% sequence identity with YhdE, overexpression of this homolog in *Bacillus subtilis* causes inhibition of cell division and extensive filamentation of cells^2^. Hence this protein is named multicopy associated filamentation (Maf) protein. Now it is known that Maf is a highly-conserved protein found in various organisms from archaea to eukaryotes, forming a large protein family. There was no further function study of Maf family proteins until crystal structures of Maf protein were determined^3^. These crystal structures exhibit 3D structure similarities with nucleotide pyrophosphatase such as Mj0226^4^ and YjjX^5^ proteins, which suggested the pyrophosphatase activity of Maf family. Subsequent enzymatic studies have identified NTPase activity for Maf proteins^6, 7^. Although lacking direct experimental evidence, it has been postulated that Maf proteins may be involved in the regulation of DNA/RNA damage repairing^8^, and are “house-cleaning”^9^ nucleotide hydrolyzing enzymes.

Overexpression of *E. coli* YhdE, a Maf family member, is also found to result in long rod shape of cell and the YhdE knockout cells seem to exhibit more spherical shape compared to wild-type cells. Furthermore, similar to other Maf proteins YhdE also possesses hydrolysis activity towards dTTP, UTP, 5-methyl-UTP and pseudo-UTP^6, 7^. Although YhdE and other Maf proteins are classified as pyrophosphatases which hydrolyze NTP to NMP as detected by high-performance liquid chromatography (HPLC)^7^, it is currently unknown whether the other hydrolysis product is phosphate (Pi) or pyrophosphate (PPi). The two scenarios would necessitate different reaction mechanisms. Direct production of Pi would suggest as a two-step sequential reaction, while formation of PPi would represent a non-sequential mechanism. Furthermore, PPi is a very important anion in biological processes in energy transduction in organisms, and it appeared to be a more favourable source of energy than ATP in early history of life^10, 11^. PPi is also involved in glycolysis^12, 13^, mitochondrial membrane potential^14, 15^, and calcium-phosphate deposition^16–18^ in different organisms. Therefore, further study of YhdE hydrolysis may provide new insights into the enzymatic mechanism of YhdE and other Maf family proteins.

PPi is unstable and can be readily hydrolyzed to Pi in acidic environment and conditions used for mass spectrometry analysis. As such, in most enzymatic assays of pyrophosphatases, PPi is not directly detected^19–22^. These methods all suffer from their indirect nature in which the amount of PPi produced cannot be directly measured. These indirect methods used to assay pyrophosphatases do not answer the question of whether PPi or Pi is produced^20, 23^. Further, the multi-step procedure and the need of various reagents including specific enzymes can be cumbersome. In recent years, highly sensitive and selective chemosensors that can quantify PPi directly are being developed. Since the pioneering work on the fluorescent sensing of PPi by Czarnik^24^ *et al*., there has been steady progress in developing PPi sensors in the past two decades. In some of the recent studies, PPi sensors have been tested to monitor PPi real-time for cell imaging^25, 26^, DNA sequencing^27^, or for real-time fluorescence assay to examine inorganic pyrophosphatase with PPi as the substrate^25, 28^. We developed a new PPi sensor, 4-isoACBA^29^, which is highly fluorescent itself but the fluorescence is quenched when two 4-isoACBA molecules coordinate with a Zn^2+^ ion. The 4-isoACBA-Zn(II) complex binds with PPi with high specificity and the fluorescence is recovered. This sensor has remarkable binding affinity and high sensing selectivity for PPi and can work in various buffer solution of neutral pH which is harmless and appropriate for most proteins such as YhdE.

Here, we reveal that PPi is the direct product of dTTP hydrolysis by YhdE using a fluorescence sensor, 4-isoACBA-Zn(II) complex. This is the first time that such a sensor is applied to detection of PPi formation for pyrophosphatase. Using the fluorescence sensor, we also show that the method is highly sensitive which can detect very weak hydrolysis activity of an YhdE active-site mutant (YhdE_E33A), which was previously shown to be completely inactive^6, 7^. We have further determined the crystal structure of YhdE_E33A and revealed the structural basis for its weak activity through a compensatory mechanism by the neighboring Glu32. Based on the crystal structure crystallized in the presence of substrate dTTP, we have carried out molecular dynamics (MD) simulation and quantum mechanics (QM) calculation to investigate the enzymatic mechanism of YhdE.

## Material and Methods

### Site-directed mutagenesis of YhdE

The mutant of YhdE was prepared using PCR with mutagenetic primers. The PCR template was the wild-type YhdE cloned in the pFO4 vector. The mutation was confirmed by DNA sequencing using the T7 promoter primer. The recombinant mutant was transformed to BL21(DE3) *E. coli* cells.

### Protein expression, crystallization and structure determination

YhdE and its mutant were expressed and purified as described before^6, 30^. The crystallization condition of the YhdE_E33A mutant included 0.1 M Tris (pH 8.0), 27% PEG 2000 MME and crystals were grown at 291 K for about two weeks. The crystals were soaked in the same crystallization buffer containing 1 mM dTTP for 2~4 hours. The diffraction data of the soaked crystals were collected on beamline BL17U at Shanghai Synchrotron Radiation Facility (SSRF), using a MAR 225 detector^31^. Data were processed with HKL3000R^32^ suite. The structure was solved by molecular replacement using the PHENIX suite^33^ which was also used for subsequent structure refinement. The graphic program Coot^34^ was used for model building and visualization.

### Enzymatic assays

Pyrophosphatase activity assays of YhdE and its mutant were performed using a new fluorescence PPi sensor (4-isoACBA-Zn(II) complex). The reaction condition for YhdE was determined to be 20 mM HEPES pH 7.0, 0.4 mM Zn^2+^, 0.1 mM 4-isoACBA at 298 K. To examine the specificity of the PPi sensor, a mixture of 0.1 mM Pi, PPi, dTTP and UTP and 0.1 μM wild-type YhdE was first tested. For the detection of the hydrolysis product, 0.1 mM dTTP (or UTP) was hydrolyzed by adding 0.1 μM YhdE for 3 minutes in the reaction condition as mentioned above. The fluorescence intensity of experiment sample and control samples were measured using a fluorescence microplate reader with the excitation and emission wavelengths of 350 nm and 426 nm, respectively^29^.

The kinetic analysis of YhdE and YhdE_E33A mutant pyrophosphatase activity using dTTP as the substrate was performed at the reaction condition as mentioned above. For the analysis of wild-type YhdE, different concentrations of substrate dTTP (0, 15, 25, 50, 75, 100, 125, 150, 175, 200, 225, 250, 275, 300 μM) were used, together with by 75 nM of the enzyme. For the analysis of YhdE_E33A mutant, the condition was as the wild-type except the concentration of this mutant was 7.5 μM. The generation of PPi was measured in real-time, monitoring fluorescence change as the sensor binds to PPi. The concentration of PPi was calculated using the standard PPi curve, which was obtained by detecting the fluorescence signal versus concentrations (10–50 mM) of Na_4_P_2_O_7_.

### Isothermal titration calorimetry (ITC)

All ITC experiments were carried out using a VP-ITC instrument (GE MicroCal) at room temperature. Both protein and dTTP solutions contained 20 mM Bis-Tris at pH 6.75 and 150 mM NaCl. The concentration of dTTP (or MnCl_2_) and proteins were 0.4 mM and 0.04 mM. Experiments were done in triplicate, 200 μL proteins were titrated for 20 times by 2 μL dTTP in each titration, with the interval time between two droplets of 300 s. The first injection peak was discarded from the isotherm, as were injection peaks without a stable baseline. The baseline and binding parameters were generated using the MicroCal Origin package.

### Docking and MM simulation and QM calculation

Substrate dTTP of YhdE was first docked into the binding pocket of crystal structure YhdE_E33A (PDB code: 4P0E) using AutoDock^35^. In the docking progress, substrate dTTP and the side chain of residues in the active site were set to be flexible. Docking simulations were performed using the AutoDock Lamarckian Genetic algorithm. Next, we placed the Mn^2+^ ion based on structures of other Maf family proteins.

The docked structure was subjected to molecular dynamics simulations by using Amber package^36^. First, we generated the amber parameter of dTTP by RESP fitting from the QM calculations. Hydrogens were added to the protein and eight Na^+^ ions were added to neutralize the system. After solvating the protein in a TIP3PBOX water box with the length between the protein and the edge of the box is at least 10.0 Å, the system was subjected to 5000 steps of energy minimization and was heated from 0 K to 310 K by 10.0 kcal mol^−1^ Å^−2^ constraint for 200 ps. Next, the heated structure was subjected to equilibration with no restrains at 310 K until the structure was equilibrated for 2 ns.

The QM model was extracted from the active site pocket of the equilibrated structure which includes the methyl-triphosphate tail of dTTP, the coordinated Mn^2+^ ion, four water molecules and other six relevant residues implicated in hydrolysis (Arg12, Arg13, Glu32, Asp70, Lys52, Lys146). All αC atoms were saturated by hydrogens atoms and fixed to avoid collapse of the cluster model during the geometry optimization. The cluster model contains 139 atoms in total and possesses a net charge of zero. The hybrid DFT method UB3LYP^37, 38^ was used for geometry optimization and transition state search; mixed basis set was used in the calculation with lanl2dz^39^ pseudopotential for manganese and 6–31 G(d)^40^ basis set for the rest of atoms. IRC calculations were performed to confirm the transition state structures. The zero-point energy (ZPE) effects correction and CPCM continuum solvation model were performed for energetic correction. All QM calculations were performed by Gaussian 09^41^ program. Structure figures were prepared using PyMOL and XYZ-Viewer.

## Results and Discussion

**Figure 1:**
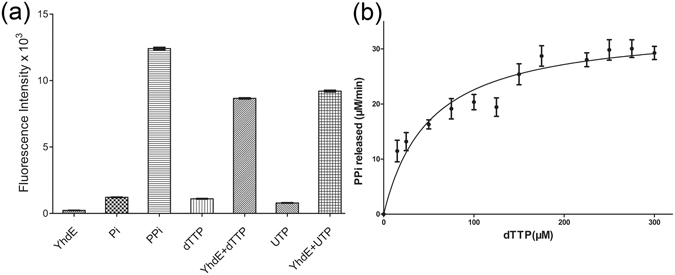
Pyrophosphatase assays of YhdE by fluorescence probe. (**a**) Fluorescence signal of 4-isoACBA-Zn(II) complex (50 μM) towards various samples in aqueous solution buffered with Hepes (20 mM, pH 7.0). Experimental conditions: 100 μM Pi, PPi, dTTP and UTP; 100 nM YhdE. (**b**) Kinetic analysis of YhdE_E33A to dTTP. For kinetic measurements, 7.5 μM YhdE_E33A was assayed under the standard conditions along with increasing concentrations of the substrate dTTP.

The fluorescence spectrum of the sensor 4-isoACBA-Zn(II) complex has very good biocompatibility and works well in our enzymatic reaction solution, which exhibits a strong emission at 426 nm when it binds with PPi upon excitation at 350 nm^29^. This new fluorescence sensor was used to determine whether the hydrolysis product of dTTP (or UTP) by YhdE is PPi or Pi. We first tested the specificity of the PPi sensor in which the fluorescence was monitored in the presence of various anions (as sodium salts). As shown in Fig. 1a, only PPi elicited significant fluorescence response, while other samples including Pi, dTTP and UTP exhibited no appreciable fluorescence response in the same conditions. We next examined YhdE hydrolysis reaction mixture in the same buffer condition and strong fluorescence signal was observed. The difference in fluorescence signal strength between YhdE reaction and the substrate (dTTP or UTP) alone without hydrolysis is ~7–12 times, clearly demonstrating the release of PPi as the direct product of YhdE-catalyzed hydrolysis of the nucleotides. This is the first time that PPi is shown to be one of the hydrolysis products of YhdE and most likely other Maf family proteins as well. Thus, the covalent bond between α-phosphoryl and β-phosphoryl of dTTP or UTP is cleaved when it is hydrolyzed by YhdE, essentially eliminating the possibility of sequential hydrolysis (releasing one phosphate at a time in two steps).

In all previous activity studies of Maf family proteins, PPi was indirectly assayed, in which PPi was further hydrolyzed to Pi by inorganic pyrophosphatase. The released Pi reacts with ammonium molybdate and malachite green to produce a colored complex^6, 7^. The coloration time of this complex can take as long as 30 minutes. In contrast, the PPi sensor can bind PPi directly and generate fluorescence instantly, which can be used for real-time analysis the pyrophosphatase activity of YhdE (Fig. S1a). Furthermore, the sensitivity is greatly enhanced. Using the indirect method, we could not detect any activity of the active site mutant in which Glu33 is changed to Ala (YhdE_E33A) and used this mutant for co-crystallization studies in a hope to form a substrate-bound complex (more see below). Interestingly, we found that the YhdE_E33A mutant still has week pyrophosphatase activity when assayed using our PPi sensor (Fig. 1b). In addition, the apparent K_m_ of YhdE_E33A binding with dTTP is 75 μM which, somewhat surprisingly, is similar to that of the wild-type YhdE. This indicates that the loss of Glu33 residue does not influence the binding affinity of YhdE for substrate dTTP. However, the apparent K_cat_/K_m_ of YhdE for dTTP is ~100 times of the YhdE_E33A mutant (Fig. 1b and Fig. S1b). The much weaker pyrophosphatase activity of YhdE_E33A which could not be detected by the conventional method suggests that the Glu33 residue plays a critically important role in YhdE’s hydrolysis activity. A main reason that conventional method involving conversion of PPi to Pi is less sensitive is because of the high Pi background. We also used this new method to detect the ITPase activity of YjjX protein and observed fluorescence signal change in the reaction mixture (data not shown), indicating that the method is generally applicable for pyrophosphatase proteins. Therefore, our new method offers a more robust and more accurate pyrophosphatase enzymatic assay than the conventional assays.

**Figure 2:**
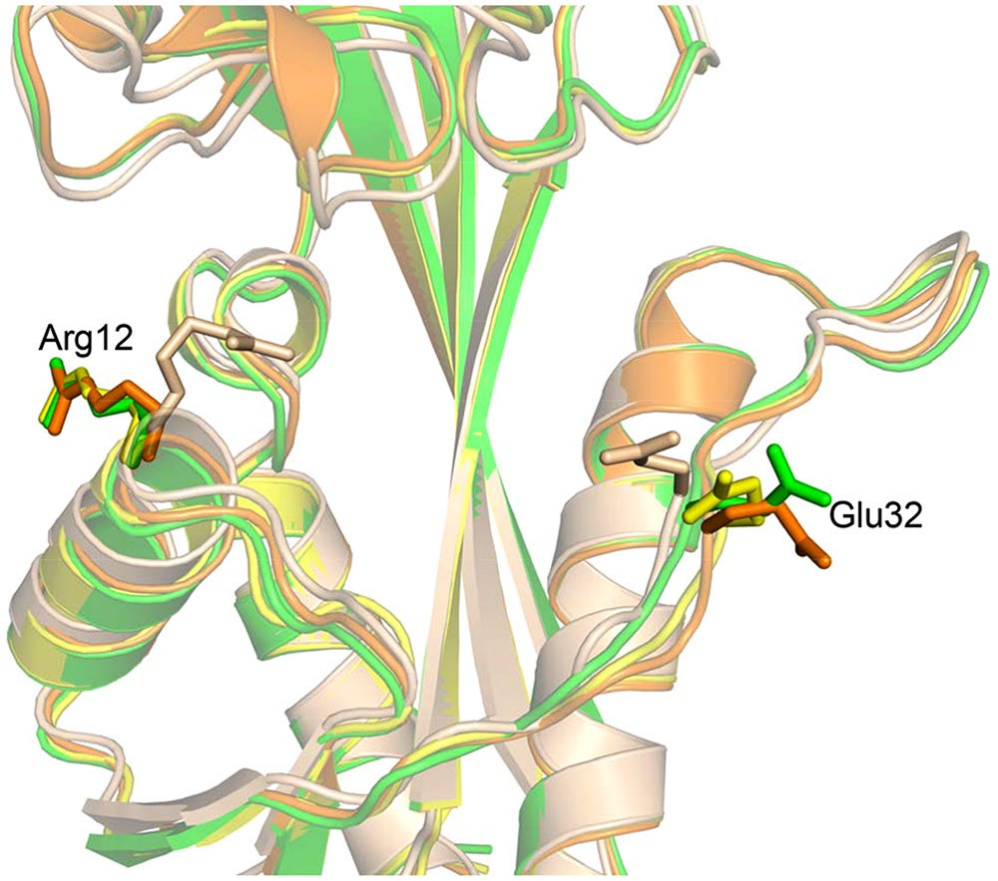
Structure superposition of four structure of YhdE_E33A. YhdE_E33A structure crystallized without dTTP, (4P0U, orange), YhdE_E33A structure co-crystallized with dTTP (4P0E, wheat), YhdE_E33A structure A soaked with dTTP (chain A, green) and YhdE_E33A structure B soaked with dTTP (chain B, yellow) are aligned together. The protein side chains are shown as sticks along with protein backbone as cartoon.

Although we were initially surprised to find that the YhdE_E33A mutant exhibits weak activity since it is known that Glu33 is critical for YhdE hydrolysis activity, we are able to rationalize the residual activity based on binding experiment and structural analysis. Enzymatic analysis by PPi fluorescence sensor shows the apparent K_m_ of YhdE_E33A with dTTP is similar to that of wild-type YhdE, which indicates that Glu33 is probably not directly involved in dTTP binding. To help understand the hydrolysis mechanism of YhdE, we previously crystallized YhdE_E33A in the absence and presence of dTTP through co-crystallization^6, 30^. Unfortunately, dTTP was not found in the YhdE_E33A structure from the co-crystallization, which is now not surprising because of the residual weak activity seen using our new PPi probe. In this work, we attempted a range of soaking experiments in which different amounts of dTTP was added, different soaking time, as well as additional dTTP using co-crystals. A representative structure of YhdE_E33A under soaking condition was determined at 2.04 Å resolution with R factor and R-free of 17.89% and 21.07% (Table S1), respectively. These efforts still did not produce a substrate-bound complex structure, although our hope was that the weak hydrolysis activity of YhdE_E33A would not be a less serious problem if sufficient amount (and/or continued supply) of dTTP is provided. Interestingly, there is subtle but potentially informative difference when the three structures of YhdE_E33A (YhdE_E33A, YhdE_E33A co-crystallization and YhdE_E33A soaking) are compared. In the structure YhdE_E33A without adding dTTP, side chains of Arg12 and Glu32 point to the opposite direction away from the active site (Fig. 2). In the co-crystalized YhdE_E33A structure, Arg12 and Glu32 rotate inwards and point to the active site. Our new soaking structure of YhdE_E33A has revealed yet another conformation in which the side chain of Glu32, but not Arg12, moves from the outside position to the inside position. This new conformation seems to represent an intermediate state between the open (no dTTP) and closed (co-crystallization) conformations. Apparently, the presence of dTTP has altered the active site conformation, exerting more influence in the solution (co-crystallization) condition than the solid state (soaking). The high B factor (Fig. S2) of Glu32 supports the notion that Glu32 is flexible and likely able to partially compensate for Glu33 which would explain the observed activity of the YhdE_E33A mutant.

**Figure 3:**
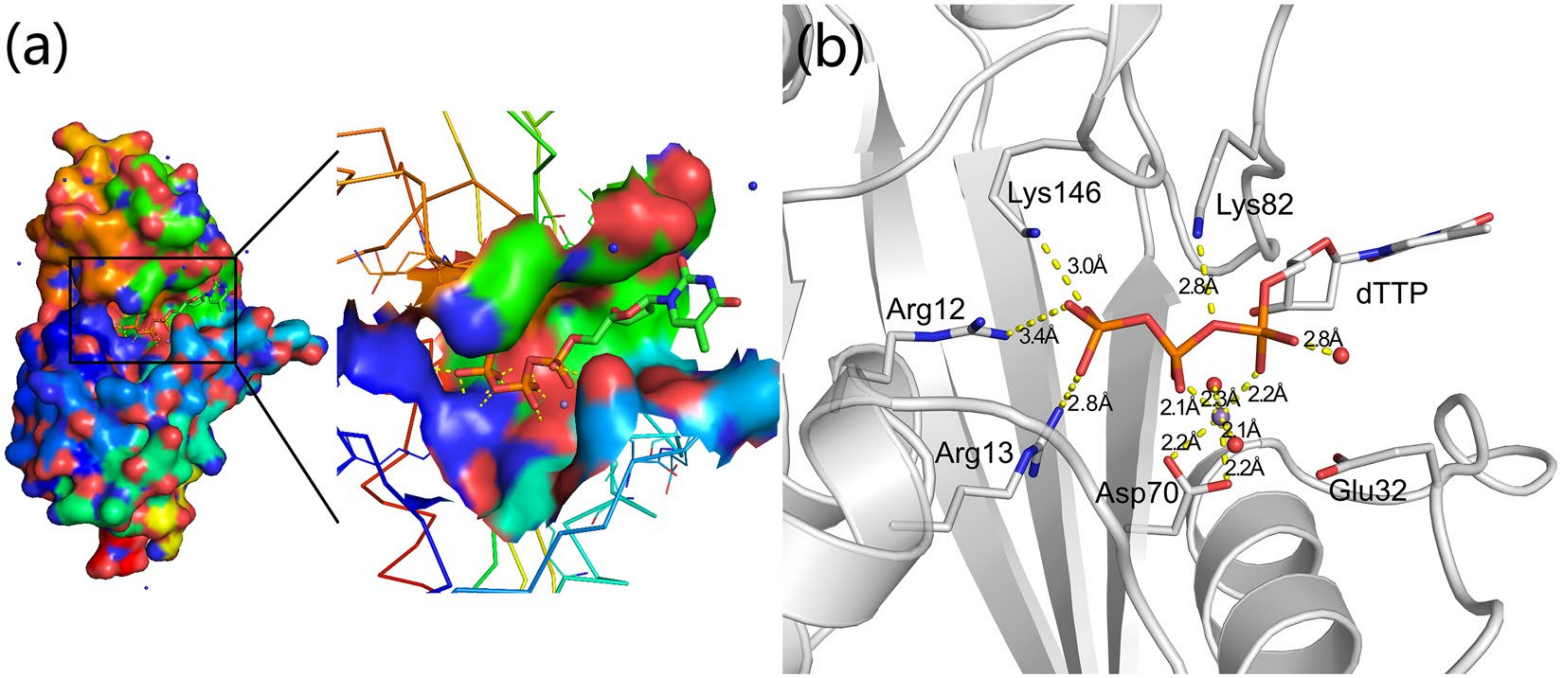
The complex structure model of YhdE_E33A and dTTP. (**a**) dTTP binding surface of YhdE_E33A. The substrate dTTP is shown as sticks, and the protein is in surface representation. The triphosphate group of substrate dTTP is buried inside the active pocket of YhdE. (**b**) The active site pocket of YhdE_E33A. dTTP and residues Arg12, Arg13, Glu32, Asp70, Lys82 and Lys146 are shown as sticks along with protein backbone as cartoon (grey). The hydrogen bonds between YhdE residues with dTTP, as well as interactions with Mn^2+^ ion are represented in dash lines (yellow). The triphosphate group of dTTP forms many hydrogen bonds with basic residues of YhdE. The carboxyl group of Asp70 together with α- and β-phosphoryl groups of dTTP and two water molecules coordinate Mn^2+^, which form an octahedral structure.

Since we were not successful in determining the wild-type YhdE crystal structure after many attempts, we elected to use YhdE_E33A mutant structure to build our initial cluster model for computational studies. There are evidences that substrate dTTP is bound in the conserved binding cleft^3, 7^. Docking approach was employed to insert dTTP to the active site of the YhdE_E33A crystal structure (PDB ID: 4P0U). The docked structure (Fig. S3) was subjected to energy minimization and MD equilibration for 2 ns before QM calculation. In the equilibrated structure from MD, the triphosphate group of the substrate dTTP is positioned in the catalytic pocket (Fig. 3a), in agreement with the structure of homologous Bacillus subtilis Maf protein (PDB ID: 1EXC). In the active site (Fig. 3b), the basic residues including Arg12, Arg13, Lys82 and Lys146 form extensive electrostatic interactions with the triphosphoryl group of dTTP. Two oxygen atoms of Asp70 and one oxygen atom from each of α-, β- phosphoryl groups along with two water oxygen atoms coordinate Mn^2+^ to form an octahedral structure, stabilizing the position of substrate dTTP and making the α-phosphorus atom of dTTP more electrophilic which can be readily attacked by a nucleophile group. There is no room for the active site pocket to accommodate an acidic residue to attack the β- or γ-phosphoryl which would generate Pi like in the case of Kinases and typical ATPase. Given the spatial position of glutamic acid residue in the active loop relative to the substrate, it can only attack the α-phosphoryl of dTTP with the help of a water molecule, yielding products dTMP and PPi. Based on these considerations, we thus built the cluster model which includes substrate dTTP, Mn^2+^ and other conserved residues which interact with the substrate and metal ion. Site-directed mutagenesis and pyrophosphatase activity analysis of YhdE^6, 7^ suggest that Glu33 may act as a proton receptor and attack the α-phosphoryl group of dTTP with the help of a water molecule. As mentioned previously, in the mutant crystal structure Glu32 takes place of Glu33 to partially retain the pyrophosphatase activity. Therefore, in our model we kept a glutamic acid (Glu32) in the active loop.

**Figure 4:**
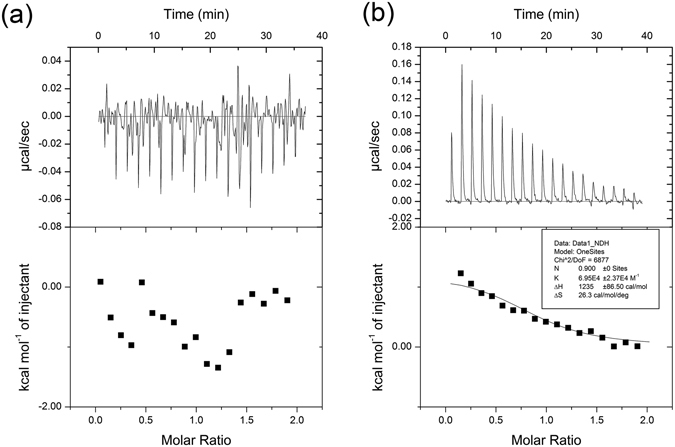
ITC experiments and interaction studies of the YhdE_D70A mutant with Mn^2+^ and dTTP. (**a**) ITC titration of 400 μM Mn^2+^ into 40 μM YhdE_D70A. (**b**) ITC titration of 400 μM dTTP into 40 μM YhdE_D70A.

As aforementioned, our model predicts that Asp70 of YhdE is a Mn^2+^-coordinating residue and may also play a modest role in the stabilization of the substrate dTTP, amongst multiple residues including the four basic amino acids. To provide experimental validation for our model, we tested the interaction between the YhdE D70A mutant with Mn^2+^ or dTTP using isothermal titration calorimetry (ITC). In the ITC experiment, wild-type YhdE protein and YhdE D70A mutant were titrated by Mn^2+^. The binding constant K_a_ of YhdE for Mn^2+^ is ~1.65 × 10^5^ M^−1^, indicating a strong interaction (Fig. S4a). In comparison, YhdE_D70A (Fig. 4a and Fig. S4b) showed no binding between the mutant and Mn^2+^. Furthermore, the binding constant K_a_ of YhdE_D70A and dTTP were similar in the presence and absence of Mn^2+^ (Fig. 4b and Fig. S4c). In agreement with our model, these results suggest that the YhdE_D70A mutation has impaired Mn^2+^ binding but is insufficient to compromise dTTP interaction with the protein. This is not surprising since dTTP is stabilized by many more interactions than Mn^2+^.

**Figure 5:**
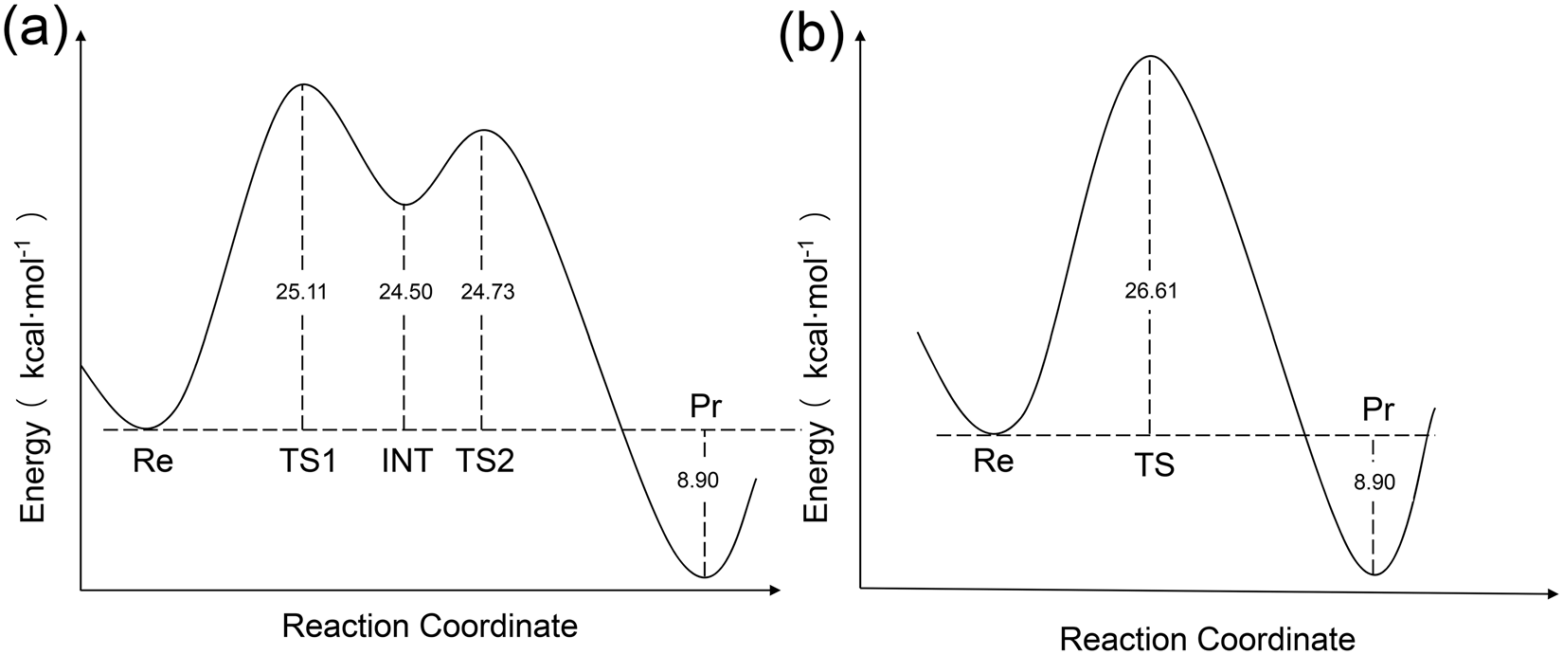
Calculated energy profile along the reaction coordinates in two YhdE hydrolysis pathways. (**a**) Stepwise pathway. The energy barrier of this pathway is 25.11 kcal mol^−1^. (**b**) Concerted pathway. The energy barrier of this pathway is 26.61 kcal mol^−1^.

**Figure 6:**
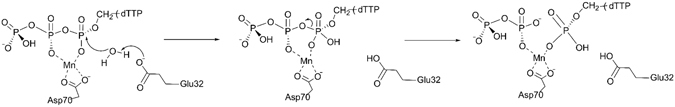
YhdE’s pyrophosphatase reaction scheme in a two-step associative pathway.

Based on the validated model and the identification of PPi as the product, we proceeded with computational studies to investigate the hydrolysis reaction mechanism by density functional theory (DFT). Two pathways of dTTP hydrolysis mechanism of YhdE were identified (Fig. 5). The first one is a stepwise pathway or, more specifically, a two-step associative pathway (Fig. S5). In this mechanism, the lytic water molecule attacks the α-phosphorus atom of the substrate dTTP in the first step, followed by the release of PPi. The α-phosphorus which is highly electrophilic as a result of coordinating Mn^2+^. The structural changes along the reaction path are shown in Fig. S5. By crossing a 25.11 kcal mol^−1^ energy barrier (Fig. 5a), Glu32 serves as a proton acceptor to extract a proton from the lytic water. Then the dTTP together with the nucleophile hydroxyl group form a pentavalent intermediate. This step is the rate-determining step of the reaction. In the subsequent step, the new P-O bond gradually becomes tighter and the old P-O bond from the opposite position is broken and releases the PPi group. The second pathway represents a concerted reaction mechanism in which the water attacks the α-phosphorus atom and PPi group is released at the same time (Fig. S6). In this process, the two P-O bonds are being broken and formed simultaneously at the top of the “energy hill”. The energy barrier of this pathway is 26.61 kcal mol^−1^ (Fig. 5b), ~1.5 kcal mol^−1^ higher than the stepwise pathway. Optimized structures along this pathway are shown in Fig. S5. Based on the computational results, we suggest that the two-step associative pathway is the favored pathway for YhdE’s pyrophosphatase reaction (Fig. 6). Besides, Mn^2+^ and the glutamic acid in the active loop play important roles in both pathways, which is consistent with our previous experiments.

## Conclusion

We have revealed that PPi is the product of YhdE hydrolysis reaction for the first time using our PPi fluorescence sensor. Based on the structures of YhdE and other Maf proteins, through MD simulation, QM calculation and PPi detection experiment we have established the enzymatic pathway. Whereas the basic residues (Arg12, Arg13, Lys82 and Lys146) in YhdE active pocket interact with triphosphate group of the substrate, Asp70 and α-, β-phosphoryl groups of the substrate coordinate Mn^2+^. A glutamic residue in the active site loop together with a water molecule attack the α-phosphorous of dTTP and generate PPi and dTMP. Given the highly-conserved nature (particularly in the active site) of Maf proteins, the reaction mechanism is most likely applicable to the entire Maf family. The precise physiological function of Maf family proteins is still not very clear and further investigation is warranted. Additionally, the role of PPi resulted from Maf protein’s NTP hydrolysis in cells is also a mystery, which may be implicated the function of Maf family proteins.

## Electronic supplementary material

Supplementary Information
