# Supplementary material for: A new method to investigate the catalytic mechanism of YhdE pyrophosphatase by using a pyrophosphate fluorescence probe
Source: Sci Rep. 2017 Aug 15;7:8169. doi: 10.1038/s41598-017-08368-1 (PMC5557916; doi:10.1038/s41598-017-08368-1)
Supplement: Supplementary file 1 — Supplementary Information [file 41598_2017_8368_MOESM1_ESM.pdf]

Supplementary Information

A new method to investigate the catalytic mechanism of YhdE pyrophosphatase by using a pyrophosphate fluorescence probe

Qingya Shen[1], Hongwei Tan[1], Guo-wen Xing[1], Jimin Zheng[1,*] and Zongchao Jia[2,*]

[1] College of Chemistry, Beijing Normal University, Beijing, 100875, China

[2] Department of Biochemical and Molecular Science, Queen University, Kingston, Ontario, K7L 3N6, Canada

* To whom correspondence should be addressed. Tel: +86-010-58806002; Email: jimin_z@bnu.edu.cn

Correspondence may also be addressed to jia@queensu.ca

Supplementary Figures

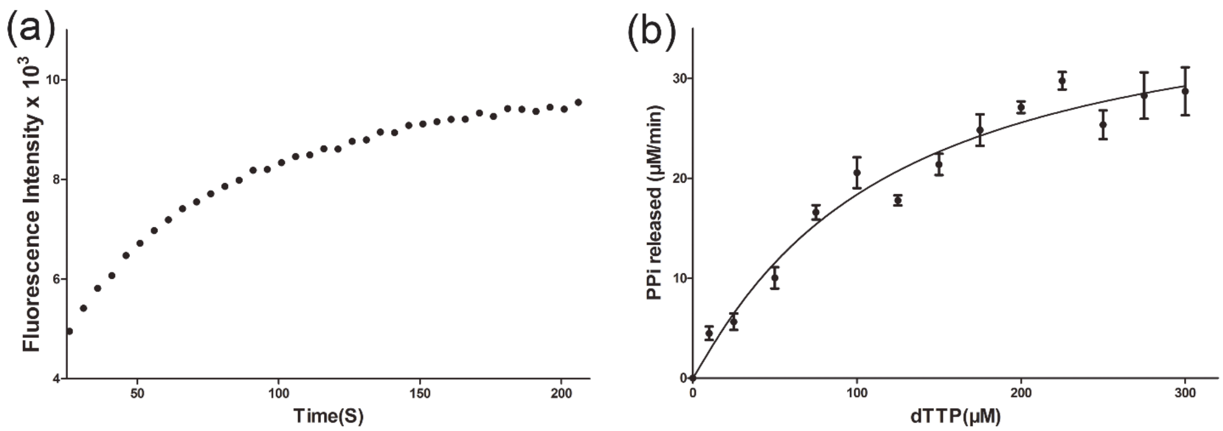

Figure S1 Pyrophosphatase assays of YhdE by fluorescence probe. (a) Real-time assays of YhdE pyrophosphatase activity by fluorescence sensor. The prior 26 seconds data could not obtained cause the response time of people and machine. (b) dTTPase activity of YhdE. For kinetic measurements, 75 nM YhdE was assayed under the standard conditions with increasing concentrations of the substrate dTTP.

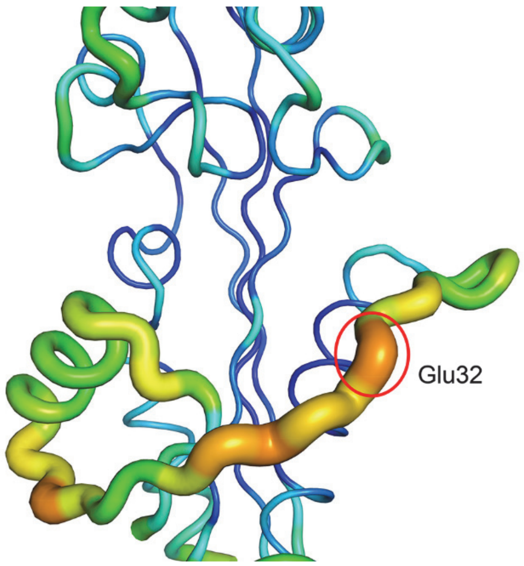

Figure S2 The B factor putty cartoon of YhdE_E33A structure soaked with dTTP (chain B). The rainbow colours from red to blue and the thickness of residues from thick to thin represent B factor from high to low. Glu32 highlighted in the red circle indicates its high B factor.

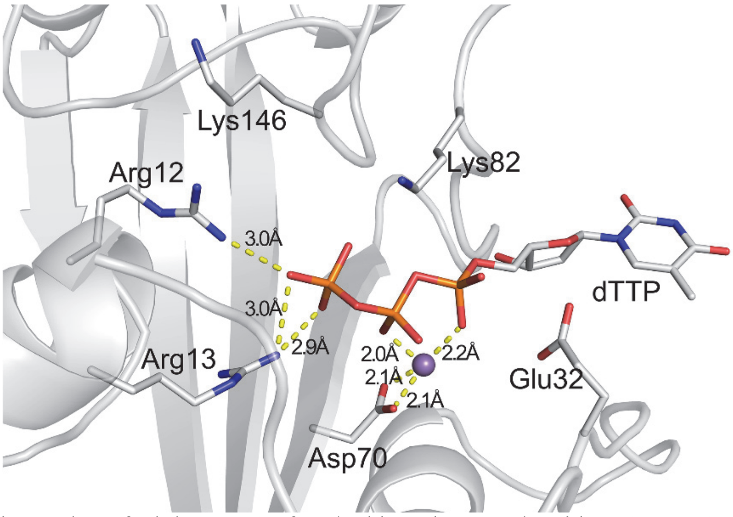

Figure S3 The active site pocket of YhdE_E33A after docking. dTTP and residues Arg12, Arg13, Glu32, Asp70, Lys82 and Lys146 are shown as sticks along with protein backbone as cartoon (grey). The hydrogen bonds between YhdE residues with dTTP, as well as interactions with Mn^{2+} ion are represented in dash lines (yellow). The triphosphate group of dTTP forms many interactions with basic residues of YhdE. The carboxyl group of Asp70 together with α- and β-phosphoryl groups of dTTP coordinate with Mn^{2+}.

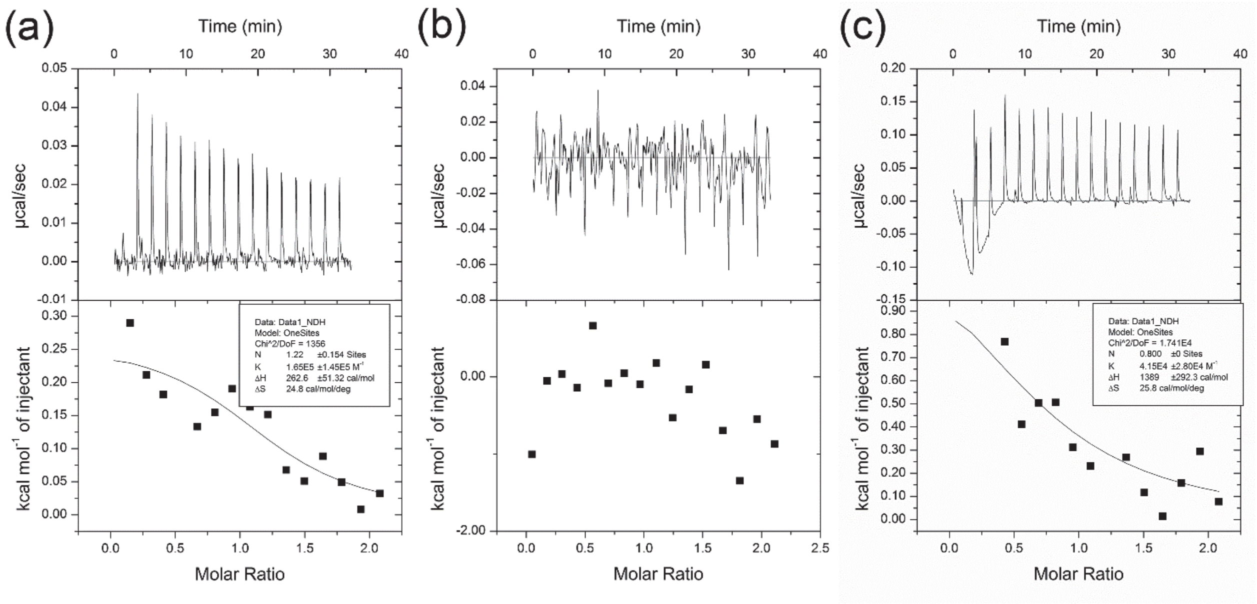

Figure S4 ITC experiments and interaction studies of the YhdE_D70A mutant with Mn^{2+}. (a) ITC titration curve of 400 µM Mn^{2+} into 40 µM wild-type YhdE as a positive control. (b) ITC titration curve of 400 µM Mn^{2+} into the buffer as a negative control. (c) ITC titration curve of 400 µM dTTP into 40 µM YhdE_D70A in the presence of 1 mM $MnCl_2$.

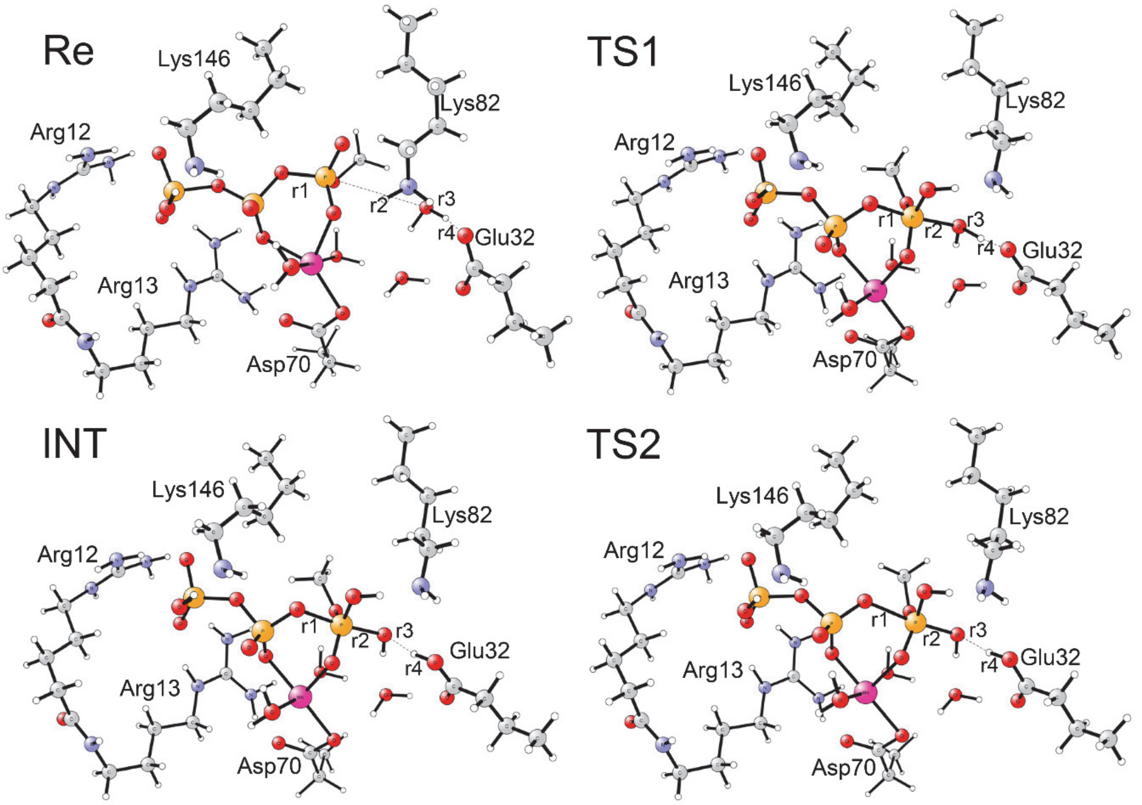

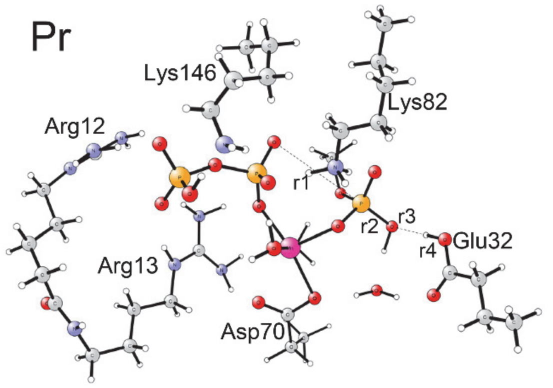

Distance(Å)	Re	TS1	INT	TS2	Pr
r1	1.65	1.77	1.85	2.14	4.24
r2	3.99	1.93	1.76	1.71	1.62
r3	1.00	1.15	1.63	1.69	1.75
r4	1.78	1.31	1.02	1.01	1.00

Figure S5 Optimized structures of Reactant (Re), Transition state 1 (TS1), Intermediate (INT), Transition state 2 (TS2) and Product (Pr) in the stepwise pathway. The important distances (Å) for various stationary reaction coordinates along the pathway are listed in the lower right table.

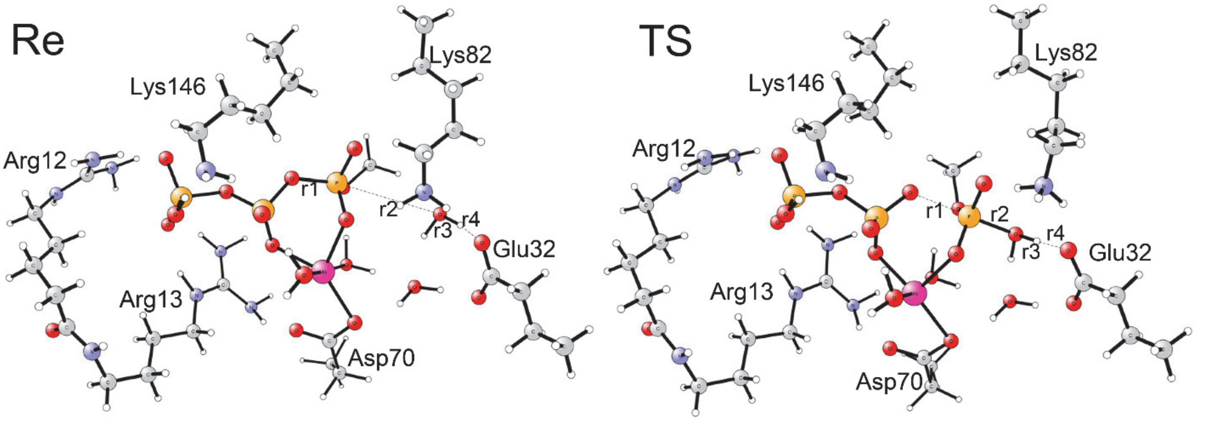

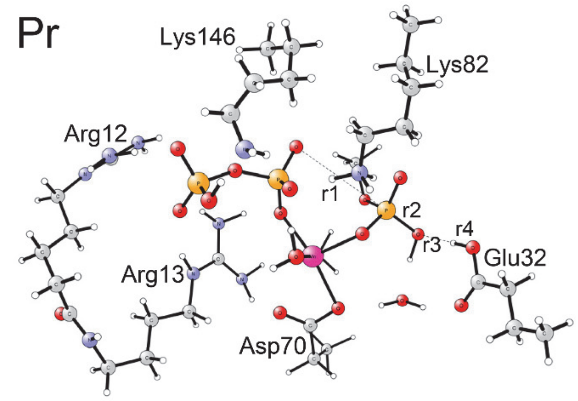

Distance(Å)	Re	TS	Pr
r1	1.65	2.29	4.24
r2	3.99	1.86	1.62
r3	1.00	1.07	1.75
r4	1.78	1.48	1.00

Figure S6 Optimized structures of Reactant (Re), Transition state (TS) and Product (Pr) in the concerted pathway. The important distances (Å) for various stationary reaction coordinates along the pathway are given in the lower right table.

Supplementary table

Table S1 Data collection and refinement statistics of YhdE_E33A crystal soaked with dTTP. Statistics for the highest-resolution shell are shown in parentheses.

Wavelength (Å)	0.97899	R-work	0.1789 (0.2135)
Resolution range (Å)	33.59 - 2.04 (2.11 - 2.04)	R-free	0.2107 (0.2631)
Space group	P 4_3	Number of non-hydrogen atoms	3181
Unit cell	71.234, 71.234 76.175; 90, 90, 90	Protein residues	373
Total number of reflections	168730	RMS (bonds, Å)	0.004
Unique number of reflections	24274 (2380)	RMS (angles, °)	0.80
Completeness (%)	99.8(100)	Ramachandran favored (%)	97
Mean I/sigma(I)	12.9(9.5)	Ramachandran allowed (%)	3
Wilson B-factor	22.83	Ramachandran outliers (%)	0
R-merge	0.071 (0.228)	Rotamer outliers (%)	1.7
R-meas	0.078 (0.245)	Clashscore	4.92
CC 1/2	0.977	Average B-factor	28.87
Number of reflections used in refinement	24273 (2380)	Number of solvent molecules	34
Number of reflections used for R-free	2033 (185)	Number of TLS groups	1
